# Appropriate Mother Wavelets for Continuous Gait Event Detection Based on Time-Frequency Analysis for Hemiplegic and Healthy Individuals

**DOI:** 10.3390/s19163462

**Published:** 2019-08-08

**Authors:** Ning Ji, Hui Zhou, Kaifeng Guo, Oluwarotimi Williams Samuel, Zhen Huang, Lisheng Xu, Guanglin Li

**Affiliations:** 1 College of Medicine and Biological Information Engineering, Northeastern University, Shenyang 110819, China; 2 CAS Key Lab of Human-Machine Intelligence-Synergy Systems of Shenzhen Institutes of Advanced Technology, Chinese Academy of Sciences (CAS), Shenzhen 518055, China; 3 School of Automation, Nanjing University of Science and Technology, Nanjing 210094, China; 4 Panyu Central Hospital, Guangzhou 511400, China

**Keywords:** gait event detection, hemiplegic gait, appropriate mother wavelet, acceleration signal, wavelet-selection criteria

## Abstract

Gait event detection is a crucial step towards the effective assessment and rehabilitation of motor dysfunctions. Recently, the continuous wavelet transform (CWT) based methods have been increasingly proposed for gait event detection due to their robustness. However, few investigations on determining the appropriate mother wavelet with proper selection criteria have been performed, especially for hemiplegic patients. In this study, the performances of commonly used mother wavelets in detecting gait events were systematically investigated. The acceleration signals from the tibialis anterior muscle of both healthy and hemiplegic subjects were recorded during ground walking and the two core gait events of heel strike (HS) and toe off (TO) were detected from the signal recordings by a CWT algorithm with different mother wavelets. Our results showed that the overall performance of the CWT algorithm in detecting the two gait events was significantly different when using various mother wavelets. By using different wavelet selection criteria, we also found that the accuracy criteria based on time-error minimization and F1-score maximization could provide the appropriate mother wavelet for gait event detection. The findings from this study will provide an insight on the selection of an appropriate mother wavelet for gait event detection and facilitate the development of adequate rehabilitation aids.

## 1. Introduction

Gait disorders are usually associated with an ageing population as well as stroke survivors with hemiplegia, thus leading to both a reduced quality of life and an increased mortality rate. The detection of gait events or gait characteristics is essential to numerous applications including the development of control mechanisms in drop foot correction devices [1,2,3,4,5], human activity recognition for healthcare [6,7], motor recovery assessments for effective rehabilitation strategies [1,8], especially for patients with lower limb motor dysfunction following a severe stroke. Heel strike (HS) and toe off (TO) are regarded as two core gait events in a normal gait cycle, which provide information on the swing, stance, and stride gait parameters. Hence, it is essential to develop an effective algorithm for the accurate detection of HS and TO gait events.

With the rapid development of wearable electronic devices, inertial sensors such as accelerometers are fast becoming widely used in gait analysis due to their portable, low-power consumption, and low-cost characteristics. In this regard, various algorithms aiming at gait event detection based on acceleration signals have been proposed in the recent years. In previous studies [4,9,10,11], a number of proposed algorithms have attempted to detect gait events based on a peak detection approach that integrates a filtering technique to the acceleration signal based on a set of predefined thresholds in the time domain. However, prior knowledge of the optimal thresholds is relatively difficult to adapt to different subjects in a real-world environment. Also, some attempts have been made to utilize machine learning algorithms for gait event detection via classification models especially with labelled datasets [12]. Note that such machine learning based approaches are limited because their operational procedures are driven by black-box models. Thus, it is difficult for developers and clinicians to truly understand the mechanism of gait event detection that often hinders proper interpretation for practical applications [9,13,14]. By exploiting the limitations of the above described threshold-based approaches and machine learning based methods [1,8], continuous wavelet transform (CWT) based methods have been considered as an alternative solution. By simultaneously providing a signal’s information in the time and frequency domains through a series of decomposition and reconstruction operation, the wavelet transform method has been utilized as an effective tool in various fields, such as signal de-noising [15], speech/signal processing and evaluation [16,17,18], and health threatening illness diagnosis [19]. In the aspect of gait research, CWT based methods have been proven to offer a time-frequency representation with the capability to capture and analyze varying frequencies of acceleration signals, be adaptive to irregular acceleration patterns even in the presence of frequency variations, as well as to attenuate inherent noise and baseline drift [20]. Considering these advantages, wavelet transforms are being increasingly used and have been reported to have good results particularly for gait event detection [13,20,21,22,23,24,25,26,27,28].

Table 1 summarizes the recently proposed CWT-based methods for detecting gait events, in which different mother wavelets were used in those previous studies. Note that the selection of an appropriate mother wavelet has been stated as a central and open question in the application of wavelet transforms for gait signal analysis [29]. Thus, finding an appropriate mother wavelet for gait events detection could be a crucial step, which would benefit coefficient reconstruction and feature extraction from the signal in time and frequency domains. Meanwhile, in other research domains such as signal de-noising, several mother wavelet selection criteria have been investigated based on some qualitative or quantitative approaches [15,17,30,31,32,33]. Apart from the fact that the quantitative method has been strongly advocated, mother wavelet selection based on accuracy driven criteria has attracted a lot of research attention in the recent years due to its simplicity and reliability [30]. However, how to determine the appropriate mother wavelet in gait event detection was rarely reported in the previous studies, so the criteria for the mother wavelet selection is still lacking. Additionally, while most of the previous studies concentrated on the gait analysis of healthy subjects and Parkinson’s disease (PD) patients, as showed in Table 1, few studies have been conducted to detect the gait events for hemiplegic patients who often suffer from gait disorders.

Towards determining an appropriate mother wavelet on gait event detection for both healthy subjects and hemiplegic patients, this study firstly constructed a general CWT algorithm. Secondly, we investigated the performance of 32 commonly applied mother wavelets in the recognition of two important gait events (HS and TO) using the acceleration signals obtained from 16 participants (including healthy subjects and hemiplegic patients) during level ground walking. By comparing the performance of different mother wavelets based on both accuracy (time-error, F1-score) and quantitative criteria (cross-correlation coefficient, energy-to-Shannon entropy ratio), the appropriate mother wavelet would be obtained. In this paper, the remaining part is organized as follows. Section 2 describes the improved general CWT algorithm and the criteria for selecting the appropriate mother wavelet. Section 3 presents the experimental results for gait event detection amongst the different mother wavelets and the different wavelet selection criteria in both healthy subjects and hemiplegic patients. Section 4 presents the discussion, and finally Section 5 concludes the work.

## 2. Materials and Methods

### 2.1. Subjects Information and Experimental Procedure

A total of 16 subjects (13 healthy subjects and 3 hemiplegic patients) participated in this study for the analysis of their gait events. The 13 healthy subjects consisted of seven males and six females with an age range of 20 to 35 years, a height of 1.55 to 1.78 m, and a weight of 47 to 78 kg. The three post-stroke patients were composed of two males and one female with an age range of 44 to 59 years, a height of 1.63 to 1.71 m, and a weight of 54 to 78 kg. For the recruitment of healthy subjects, a preliminary assessment was conducted to ensure that they had no physical problems that might affect their gait patterns. For the recruitment of hemiplegic patients, the following four inclusion criteria were ensured: They had a unilateral hemiplegia after stroke, were able to walk at least 10 m independently without any help or assistive devices after motor rehabilitation treatment, were not diagnosed with other diseases that might affect their walking patterns, and were able to understand and cooperate with the experimental protocols. With these criteria, three patients who have been diagnosed with cerebral ischemic stroke were recruited from Guangzhou Panyu Central Hospital. Each patient’s lower extremity motor functional level was evaluated using the Brunnstrom stage assessment method, which was used to reflect post-stroke motor recovery stage in clinical settings [34]. The clinical and demographic details of the hemiplegic subjects are summarized in Table 2. All the subjects gave the written informed consent to participate in the study and also provided the permission for the publication and educational purposes of their data/photographs. The experimental protocol of the study was approved by the Ethics Committee for Human Research, Shenzhen Institutes of Advanced Technology, Chinese Academy of Sciences.

**Figure 1:**
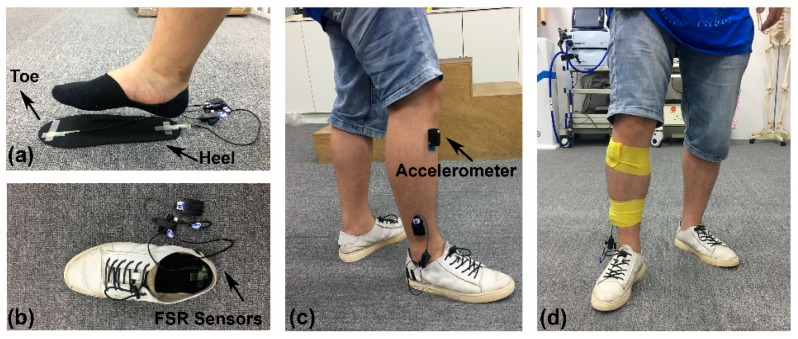
Experimental setup for force sensitive resistor (FSR) and accelerometer sensors placement in preparation for gait data acquisition. (**a**) A pair of FSR sensors were placed under the big toe and heel of the insole; (**b**) the placement of the insole into the shoe; (**c**) the placement of the accelerometer on the tibialis anterior muscle under the knee joint of right leg; (**d**) a bandage was used to firmly fix the sensors.

In the experiments, a wireless tri-axial accelerometer sensor (Delsys Inc., Natick, MA, USA) was attached to the tibialis anterior muscle of the right leg of each healthy subject and the paretic leg of each hemiplegic patient. As a reference for the ground true values of HS and TO, two force sensing resistor (FSR) sensors (Delsys Inc., Natick, MA, USA) were used to record the foot-switch signals of the right foot for comparison. To be precise, the two FSR sensors were placed under the toe and heel of each subject, respectively. The FSR and acceleration signals were concurrently recorded using a commercial data acquisition system (Delsys Inc., Natick, MA, USA) and transmitted wirelessly to a computer system for storage. To ensure high quality signal recordings, a bandage (Kindmax Inc., Irvine, CA, USA) was used to firmly fix the sensors to the leg so as to minimize their displacement and vibration during walking. A diagram showing the experimental setup is presented in Figure 1. During the experiments, each subject was instructed to walk continuously along a 10 m pathway on level ground at their preferred speed (regarded as the norm). The subjects repeatedly walked from one side of the pathway to another side, and then made a U-turn to walk back for around one minute. Both the FSR and acceleration signals were simultaneously sampled at a rate of 148.15 Hz and then the acquired signals were analyzed using an offline MATLAB R2018b programming tool.

### 2.2. CWT Based Gait Event Detection Algorithm

A cycle of human gait can be divided into a sequence of repeated events and phases. More specifically, one gait cycle consists of stance and swing phases. The HS and TO events mark the beginning of stance and swing phases, respectively. In this case, it is obvious that the occurrence frequency of the two gait events (HS and TO) is twice of one gait cycle. This time-frequency relationship between the gait event and the gait cycle through the CWT can be used to detect gait events [13]. Moreover, it should be noted that the human gait is chaotic in nature in the real-world environment [35]. Thus, continuous wavelet transform methods have been proven to be robust and stable in several previous studies in effectively detecting gait events in practical applications that may be subject to various disturbances [13,20,21,22,26,28]. Here we propose a general CWT algorithm for the detection of two gait events (HS and TO), which is a combination of the methods in the two previous studies by Minh H. Pham et al. [22] and Siddhartha Khandelwal et al. [13].

**Figure 2:**
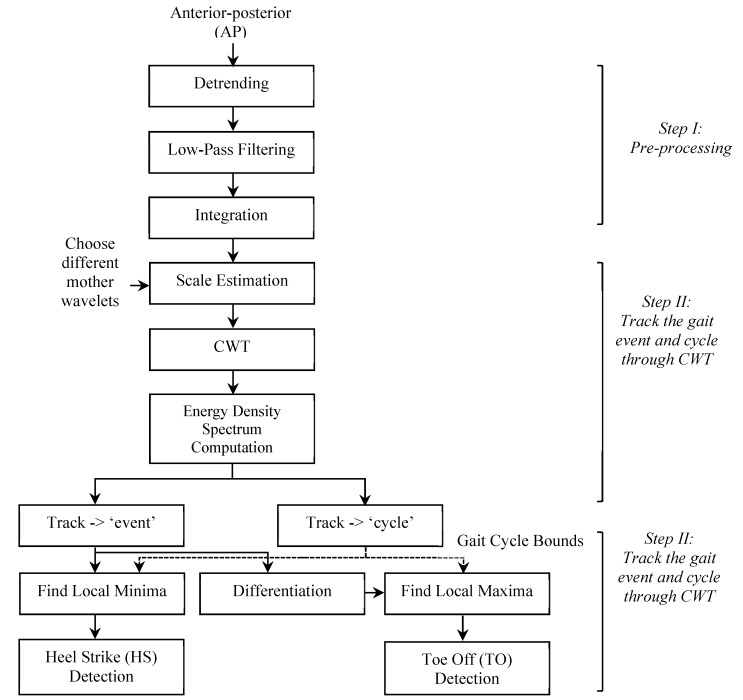
Conceptualization of the proposed general CWT algorithm with three main phases: (1) Pre-processing of the acceleration signals; (2) Tracking the gait events and gait cycles through time-frequency analysis; (3) Distinguishing HS and TO gait events.

The operational procedure of the general CWT based gait event detection algorithm is presented in Figure 2. Here the components of the tri-axially recorded acceleration signals from the anterior-posterior (AP) axis during level ground walking were used for the analysis of gait events [22,36]. In order to improve the quality of the AP signal recordings, the algorithm began with a pre-processing of the AP acceleration signals that were composed of three sequential procedures. Firstly, a linear de-trending algorithm was adopted to reduce the effect of noise and interference on the AP signal baseline. Secondly, a low-pass filtering at 10 Hz with a second-order Butterworth filter was used to remove the high-frequency interferences from the AP signals. Finally, the AP signals were smoothed by integration using the inbuilt function in MATLAB called *cumtrapz*, where the approximation of the cumulative integral of each sampling points along the time intervals was calculated.

After the AP acceleration signal preprocessing, the time-frequency analysis was performed to track the inherent gait events as well as gait cycles. The CWT of a discrete time signal $x_{n}$ with equal time spacing $\delta_{t}$ is defined as the inner product of $x_{n}$ with a scaled and translated mother wavelet ψ as expressed in Equation (1).

(1) $$W_{n}\left(s\right)\;=\;\overset{N-1}{\underset{n^{\prime }=0}{\sum }}x_{n^{\prime }}\psi *\left[\frac{\left(n^{\prime }-n\right)\delta_{t}}{s}\right]\;\;\;$$

where $W_{n}\left(s\right)$ denotes the wavelet transform, s is the wavelet scaling factor, n is the localized time index, and the (*) indicates the complex conjugate. Detailed definitions of different mother wavelets (ψ) and the prescription of wavelet selection criteria are presented in Section 2.4. The range of scales for CWT analysis [1, $s_{max}$] was chosen using the frequency scale relationship of the chosen wavelet [26], which is presented in Equation (2).

(2) $$s_{max}\;=\;\frac{f_{c}\times F_{s}}{f}$$

where $f_{c}$ is the central frequency of the wavelet, $F_{s}$ is the data sampling frequency, and f is the gait frequency. Since the healthy subjects usually walk at an average speed of 1.5 m/s, a minimum gait frequency of 0.5 Hz was utilized in this study as the norm [26]. In the experiments, the hemiplegic subjects walked with an average speed of around 0.7–0.8 m/s. Thus, a minimum gait frequency of 0.25 Hz was assumed for the hemiplegic subjects.

**Figure 3:**
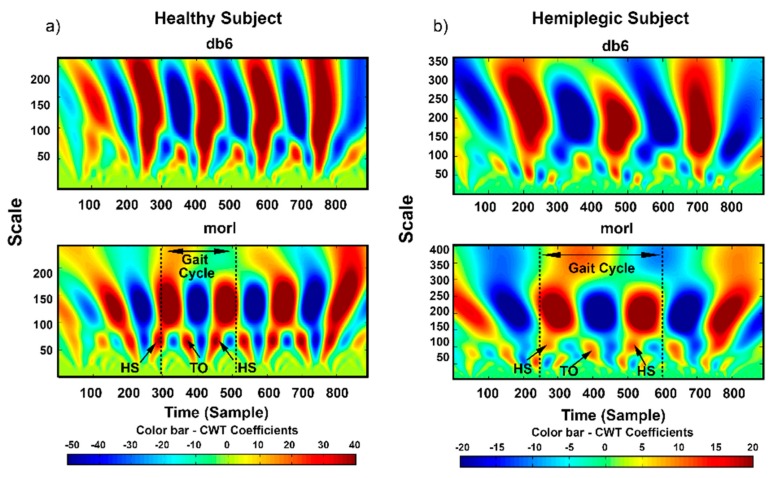
Continuous wavelet transform (CWT) plots of the acceleration signals during walking from one healthy subject (**a**) and one hemiplegic subject (**b**), where two mother wavelets (“db6” and “morl”) were adopted for comparison. The underlying scale relationship between the gait events (HS and TO) and gait cycles are illustrated.

Given a mother wavelet, the corresponding frequency scale can be calculated by Equation (2) for CWT analysis, and then the CWT coefficients can be obtained by Equation (1). As a typical example, Figure 3 graphically demonstrates the CWT plots of the acceleration signals during walking from one healthy subject (Figure 3a) and one hemiplegic subject (Figure 3b), where two mother wavelets (“db6” and “morl”) were adopted for comparison. It was obvious from Figure 3 that the frequency of gait events underlying the corresponding scales is approximately two times of gait cycles. To distinguish the gait event and the gait cycle, the scale-dependent energy density spectrum $E_{s}$ was computed based on the obtained CWT coefficients by using Equation (3).

(3) $$E_{s}\;=\;\overset{N-1}{\underset{n=0}{\sum }}{\left|W_{n}\left(s\right)\right|}^{2},\;\;\;\;\;s\in \left[1,\;\;s_{max}\right]\;\;\;\;\;$$

where N is the total number of wavelet coefficients and ${\left|W_{n}\left(s\right)\right|}^{2}$ is the 2-D wavelet energy density function that measures the total energy distribution of the signal associated with the s scale. The peaks in $E_{s}$ represent the dominant energy scales that contribute most to the signal’s energy in the spectral domain [13]. Typically, two distinct spectral energy peaks can be obtained from the above computed $E_{s}$ parameter and the corresponding peak scales have a ratio of two due to the frequency relationship between the gait event and the gait cycle. Since the candidate scales of gait events and gait cycles were found from the peaks of $E_{s}$, the peaks were stored as the set of local maxima points {($s_{m}$,$\;E_{sm}$),  m∈[1, M]}; where $s_{m}$ is the scale corresponding to a peak value of $E_{sm}$ and M is the total number of peaks. Afterwards, the following three possible cases were checked to determine the scale of the associated gait cycle and gait event in the energy density spectrum.

Case I: If there was only a single peak (M = 1) in the energy density spectrum, then the dominant spectral scale was tracked as the cycle scale using $s_{cycle}=s_{1}$ and the event scale was tracked using the $s_{event}\;=\;round\left(s_{cycle}/2\right)$ relationship.

Case II: If there were two dominant peaks (M = 2) in the energy density spectrum, then the cycle scale was tracked using $s_{cycle}=s_{2}$ and the event scale was tracked using the $s_{event}=s_{1}$($s_{1}<s_{2}$) relationship.

Case III: If there were more than two peaks (M ≥ 3) in the energy density spectrum, then the ratio of any two consecutive (neighboring) peaks denoted as $\delta \;=\;s_{m+1}/s_{m}$ ($s_{m}<s_{m+1},m\in \left[1,M\right))$ was checked with the relaxed spectral relationship that δ ∈ [1.6, 2.4] to attenuate interferences in the signal and manage the occurrence of low frequency [14]. Thus, the two latest peaks that satisfied the above condition were tracked as the dominant peaks using the $s_{cycle}=s_{m+1}$ and $s_{event}=s_{m}$ ($s_{m}<s_{m+1}$) relationships.

Based on the conditions specified in Cases I–III, the scales of gait event and gait cycle were determined with the associated CWT coefficients ($x_{event}$ and $x_{cycle})$ as shown in Equations (4) and (5).

(4) $$x_{event}≜\;W_{n\;}\left(s_{event}\right)$$

(5) $$x_{cycle}≜\;W_{n}\;\left(s_{cycle}\right)$$

**Figure 4:**
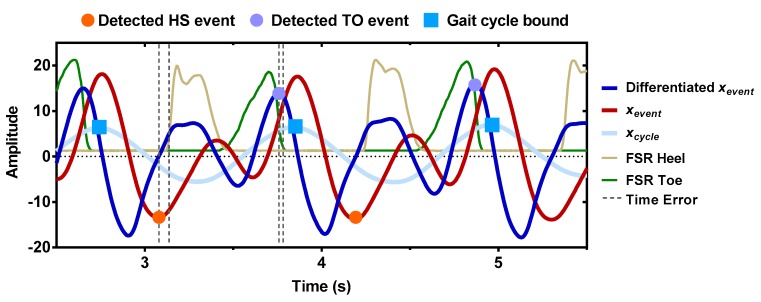
Temporal representation of HS and TO gait event detection based on the proposed general CWT algorithm. The first local minima of $x_{event}$ corresponded to the estimated HS event (orange circle). The second local maxima of the further differentiated $x_{event}$ corresponded to the estimated TO event (purple circle). Each gait cycle bound was defined as the maximum points of $x_{cycle}$. Vertical dashed lines indicated the time-errors between the estimated event and the reference event from the FSR method.

After de-trending both the above temporal signals ($x_{event}$ and $x_{cycle}\;$), the gait cycle bounds could be defined as the maximum points of $x_{cycle}$, which marked the beginning of each gait cycle. Within every gait cycle, the HS event was defined as the first local minima of $x_{event}$ and TO event was defined as the second local maxima of the further differentiated $x_{event}$. An illustration of the temporal representation of HS and TO gait event detection based on the proposed general CWT algorithm is illustrated in Figure 4.

### 2.3. Verification of the CWT Based Gait Event Detection Algorithm

As a direct measure of pressure distribution under the foot during walking, the FSR method is commonly used as the gold standard for gait event detection in previous gait literatures [8]. Thus, the FSR method was utilized as the reference method to evaluate the performance of the proposed CWT based gait event detection method in our study. In the FSR method, the HS and TO events were detected by a set of threshold values. The threshold value for the detected HS event was calculated as 5% of the maximum heel FSR amplitude at the foot force increasing stage. Meanwhile, the threshold value for the detected TO event was calculated as 5% of the maximum toe FSR amplitude at the foot force decreasing stage. Detailed description is provided in previous literatures [26,37]. It should be noted that the heel and toe FSR signals of each subject were collected simultaneously with the walking acceleration signals through a pair of FSR sensors, which was described in the experimental procedure (see Section 2.1).

In this study, the time-error and F1-score were utilized as the metrics to assess the gait event detection performances, which were commonly adopted in the previous gait literatures [13,20,38]. It is worthy to note that the gait events detected with the FSR recordings were used as the reference events for each subject. And the gait events detected by using the proposed general CWT method based on the AP acceleration signals were designated as the estimated events. Time-error, a measure of the time agreement between the reference events and the estimated events, was utilized to assess the time accuracy among the correctly detected gait events when the proposed method was applied. The time-errors of the estimated HS and TO gait events, designated as $TE_{HS}$ and $TE_{TO}$, respectively, were defined as the following Equations (6) and (7):

(6) $$TE_{HS}=\left|T_{HS\_CWT}-T_{HS\_FSR}\right|$$

(7) $$TE_{TO}=\left|T_{TO\_CWT}-T_{TO\_FSR}\right|$$

where $T_{HS\_CWT}$ and $T_{TO\_CWT}$ represent the time indexes of the estimated HS and TO gait events via the CWT-based algorithm, and $T_{HS\_FSR}$ and $T_{TO\_FSR}$ represent the time indexes of the reference HS and TO gait events via the FSR method, respectively.

In cases of possibly missed and wrongly detected gait events, the F1-score was adopted as a measure of the precision and recall of the gait event detection, which is calculated as follows:

(8) $$F1\;=\;\frac{2PR}{P+R}$$

where P denotes Precision and R denotes Recall, which are computed as Equations (9) and (10), respectively:

(9) $$P\;=\;\frac{TP}{TP+FP}$$

(10) $$R\;=\;\frac{TP}{TP+FN}$$

where TP denotes the true positives, FN denotes false negatives, and FP denotes false positives. Note that in the context of this study, TP actually represented the number of correctly estimated gait events, FN denoted the number of unidentified/missed gait events, and FP represented the number of wrongly estimated gait events.

### 2.4. Appropriate Mother Wavelet Selection

Considering the fact that mother wavelet selection plays an important role in the overall performance of CWT based algorithms, there is a need for an effective means of determining the appropriate mother wavelet especially in the context of gait event detection for hemiplegic patients. In order to determine an appropriate mother wavelet, we systematically investigated the performance of 32 commonly applied mother wavelets in detecting gait events using the measures of both accuracy and quantitative criteria. The targeted mother wavelet functions included ten Daubechies (db1–db10), five Coiflets (coif1–coif5), seven Symlets (sym2–sym8), eight Gaussian (gaus1–gaus8), one Morlet (morl), and one Meyer (meyr). Table 3 briefly summarizes the definitions and main properties of the investigated wavelet families in this study.

Generally, the basic properties of each mother wavelet, such as the orthogonality and symmetry, are considered in order to help select the appropriate mother wavelet in the first step. The orthogonality characteristics can ensure that the signal will not be decomposed into overlapping sub-frequency bands. Symmetry property can help avoid phase distortion, which is mainly concerned in the wavelet-based filtering operation because the mother wavelet can be served as a linear phase filter. As pointed out in [30], there is possibly more than one mother wavelet sharing similar fundamental properties. Thus, the selection of an appropriate mother wavelet cannot solely rely on the basic properties of the wavelets. Besides, the visual similarity generally applied in the optimal mother wavelet selection is also reported to be not always proper for all wavelet-based processing [30,31]. Additionally, the wavelet selection that is based on accuracy related to specific application is more recommended [30]. In this case, both accuracy (time-error, F1-score of the gait event detection) and quantitative (cross-correlation coefficient, energy-to-Shannon entropy ratio) criteria were investigated to search for an effective mother wavelet associated with HS and TO event detection for healthy and hemiplegic subjects. For the accuracy criteria, kindly note that the definition of time-error and F1-score can be referred in Section 2.3.

For the quantitative criteria, cross-correlation coefficient (*Xcorr*) and energy-to-Shannon entropy ratio (*ESER*) metrics were applied to evaluate the performances of the 32 mother wavelets in gait event detection in this study. Note that they were two commonly used quantitative wavelet selection criterion in various applications such as signal de-noising, signal processing/decoding, and vibration signal analysis [15,17,39]. In the cross-correlation measure, the cross-correlation coefficient (XCorr) between the recorded acceleration signal (X) and a specific mother wavelet ($Y_{i}$) was obtained and utilized to quantify the performance of the mother wavelet ($Y_{i}$) in detecting the associated gait events [31,32]. It is noteworthy that the higher the absolute $XCorr\left(X,Y_{i}\right)$, the stronger the correlation will be and a value of 0 indicates that the two variables are linearly independent. The cross-correlation coefficient $XCorr\left(X,Y_{i}\right)$ is computed using the formula in Equation (11).

(11) $$XCorr\left(X,Y\right)\;=\;\frac{\sum^{\text{​}}\left(X-\bar{X}\right)\left(Y-\bar{Y}\right)}{\sqrt{\sum^{\text{​}}{\left(X-\bar{X}\right)}^{2}{\left(Y-\bar{Y}\right)}^{2}}}$$

In the energy-to-Shannon entropy ratio (ESER) measure, the goal is to obtain a mother wavelet that provides the maximum energy, and at the same time minimum Shannon entropy associated with the wavelet coefficients [39]. Thus, the energy-to-Shannon entropy ratio is computed using the formula in Equation (12).

(12) $$ESER\left(s\right)\;=\;\frac{E\left(s\right)}{S_{entropy\;}\left(s\right)}$$

where the energy E(s) associated with s scale was computed using Equation (3) and the Shannon entropy $S_{entropy\;}\left(s\right)$ was defined as the Equation (13).

(13) $$S_{entropy\;}\left(s\right)\;=\;-\overset{N}{\underset{i=1}{\sum }}p_{i}\cdot \log_{2}p_{i}$$

where $p_{i}$ is the energy probability distribution of each wavelet coefficient and is computed using the formula in Equation (14). $W_{n}\left(s\right)$ represents the wavelet transform of the signal associated with the s scale, which was defined in Equation (1). $E_{s}$ represents the energy density spectrum which is computed based on the above mentioned CWT coefficients $W_{n}\left(s\right)$ and was defined in Equation (3). N is the total number of wavelet coefficients. Specifically, if $p_{i}$ = 0, then $\overset{N}{\underset{i=1}{\sum }}\;p_{i}$ = 1.

(14) $$p_{i}\;=\;\frac{{\left|W_{n}\left(s\right)\right|}^{2}}{E\left(s\right)}$$

In summary, the mother wavelet that provides the maximum F1-score, XCorr, and ESER as well as the minimum time-error is generally considered to be the most appropriate mother wavelet for gait event detection.

### 2.5. Statistical Analysis

Statistical tests were conducted using a SPSS 21 software package to further validate the findings of the study. Specifically, the most appropriate mother wavelets among the healthy and hemiplegic subjects were determined by evaluating the total time-error, F1-score, XCorr, and ESER of the HS and TO events between the proposed general CWT algorithm and the FSR method through one-way ANOVA. Afterwards, a post-hoc comparison was performed based on Duncan’s test, and the significance level was set to *p* < 0.05 for all the analysis.

## 3. Results

### 3.1. Appropriate Mother Wavelet Selection Based on Accuracy Criteria

**Figure 5:**
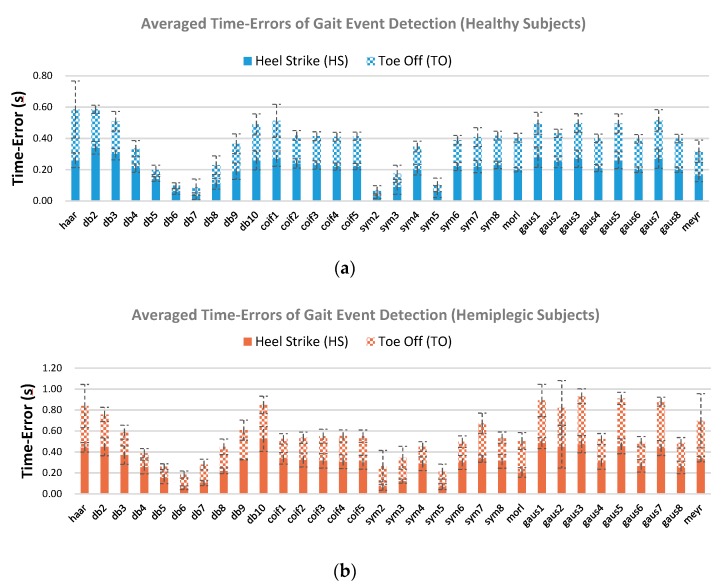
Averaged time-error values of the estimated HS and TO gait events over all the healthy subjects (**a**) and all the hemiplegic subjects (**b**) when using 32 commonly applied mother wavelets. The vertical dashed lines indicate the standard deviation.

The gait event detection results for the healthy and hemiplegic subjects using the two accuracy criteria, the time-error and F1-score measures for mother wavelet selection, are presented as follows. Figure 5 presents the averaged time-errors associated with HS and TO estimated gait events for the healthy and hemiplegic subjects across the 32 mother wavelets. Notably, the lower the time-error value is, the higher the time-agreement with the FSR reference is. It can be seen from Figure 5a that the “sym2”, “db7”, and “db6” mother wavelets achieved a relatively lower averaged time-error value of 0.06 ± 0.03 s, 0.09 ± 0.07 s, and 0.10 ± 0.03 s, respectively, for the total of the estimated HS and TO gait events over all the healthy subjects in comparison to the other mother wavelets. Meanwhile, “db6”, “sym5”, and “db5” achieved a lower averaged time-error value of 0.18 ± 0.05 s, 0.21 ± 0.07 s, and 0.26 ± 0.07 s for gait event detection over all the hemiplegic participants, respectively, as shown in Figure 5b. Note that using the “db6” mother wavelet, the averaged time-error values were lowest over the hemiplegic participants (Figure 5b) and were relatively lower over the healthy subjects (Figure 5a). These findings suggest that the “db6” is an appropriate mother wavelet in gait event detection for both healthy and hemiplegic subjects.

**Figure 6:**
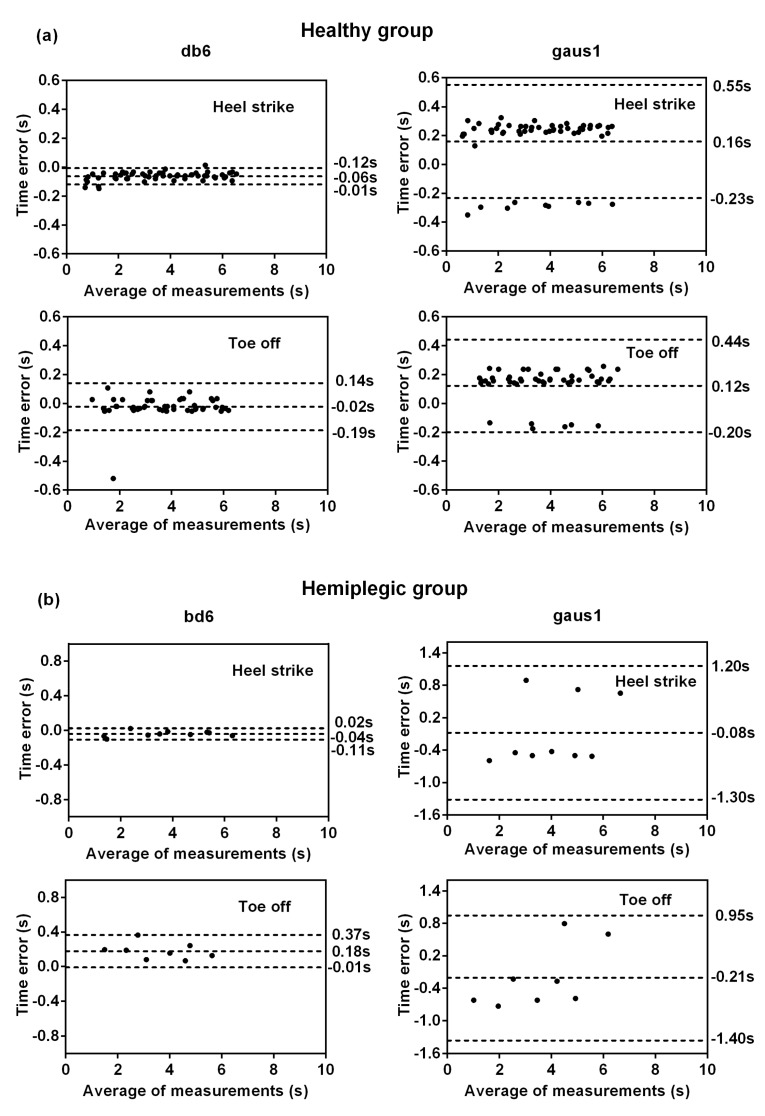
Bland–Altman plots of time agreement between the proposed modified CWT algorithm and the FSR method for HS and TO gait event detection in the healthy group (**a**) and the hemiplegic group (**b**). The time agreement results of selecting the optimal wavelet “db6” are shown on the left side whereas results of the commonly used wavelet “gaus1” with rather poor performance are shown on the right side. Positive times correspond to delays in the gait event detection of the proposed modified CWT algorithm with respect to the reference FSR method. The horizontal axis represents the average time measures for detecting gait events by both methods, and the vertical axis is the time error between the two methods. The dashed line from top to down respectively represent the 95% CI upper limit, the mean, 95% CI lower limit of the time difference (in seconds).

As a comparison, Figure 6 plots the Bland–Altman time agreements between the reference gait events and the estimated gait events, where the sample points were obtained from the time-error values of the healthy (Figure 6a) and hemiplegic subjects (Figure 6b) when using the appropriate mother wavelet “db6” and one previously used wavelet “gaus1” in another study [15], respectively. Note that the absolute value of time-errors is presented in Figure 5, whereas the true value of the time agreement is plotted in Figure 6. Here a negative time-error value corresponds to a time-delay in the estimated gait events with respect to the FSR reference gait events, and a positive value means a time-advance. We can see from Figure 6 that for the healthy subjects, the mother wavelet “db6” had a mean time-error of −0.06 s with a 95% confidence interval (CI) of [−0.12, −0.01] for HS gait event detection and −0.02 s [−0.19, 0.14] for TO gait event detection. While using the mother wavelet “gaus1”, the mean time-error of 0.16 s [−0.23, 0.55] was achieved for HS gait event detection and 0.12 s [−0.20, 0.44] for TO gait event detection. For the hemiplegic subjects, the “db6” mother wavelet achieved a mean time-error of −0.04 s [−0.11, 0.02] for HS gait event detection and 0.18 s [−0.01, 0.37] for TO gait event detection, whereas the “gaus1” mother wavelet achieved a mean time-error of −0.08 s [−1.30, 1.20] for HS gait event detection and −0.21 s [−1.40, 0.95] for TO gait event detection. Thus, the performances of “db6” were rather good in comparison to “gaus1” for both the healthy and the hemiplegic subjects.

**Figure 7:**
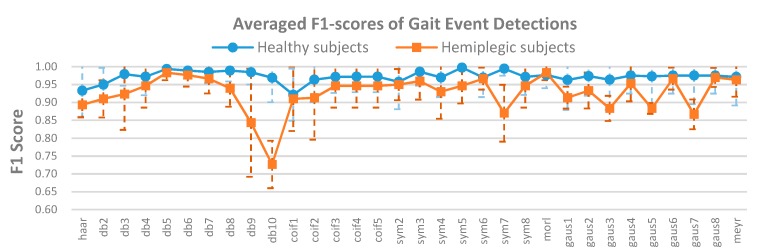
Averaged F1-scores of HS and TO gait event detection over all the healthy subjects (blue line) and over all the hemiplegic subjects (red line) across different mother wavelets. The vertical dashed lines indicate the standard deviation.

Figure 7 shows the averaged HS and TO gait event detection results obtained by using the F1-score criterion across all the 32 mother wavelets. The vertical dotted line indicates the standard deviation. It can be observed from Figure 7 that four of all the mother wavelets, “sym5”, “db5”, “db6”, and “sym7” achieved relatively higher average F1-scores (1.00 ± 0.01, 0.99 ± 0.02, 0.99 ± 0.02, and 0.99 ± 0.02 across the healthy subjects (blue line), respectively). For the hemiplegic patients (red line), “db5”, “db6”, and “morl” mother wavelets achieved higher averaged F1-scores (0.98 ± 0.02, 0.98 ± 0.03, and 0.98 ± 0.02, respectively). Note that “db5” and “db6” mother wavelets are more stable with relatively high F1-scores for both the healthy and hemiplegic subjects, indicating that the two mother wavelets would be the appropriate mother wavelets for the accurate detection of gait events in comparison to the other mother wavelets regardless of the status of the subject.

Furthermore, when using the different mother wavelets, the ANOVA analysis of the time-errors (Figure 5) between the FSR reference events and the estimated gait events was significantly different (*p* < 0.001) for both healthy and hemiplegic subjects. This statistical result suggests that the time-error should be a proper criterion for selection of an appropriate mother wavelet in gait event detection for all the subjects. Meanwhile, the ANOVA analysis of the F1-scores (Figure 7) was significantly different for the hemiplegic subjects (*p* < 0.01) and was not significantly different for the healthy subjects (*p* > 0.05). This suggested that the F1-score could also be used as a criterion for the selection of an appropriate mother wavelet in gait event detection for hemiplegic subjects, but probably not for healthy subjects.

### 3.2. Appropriate Mother Wavelet Selection Based on Quantitative Criteria

**Figure 8:**
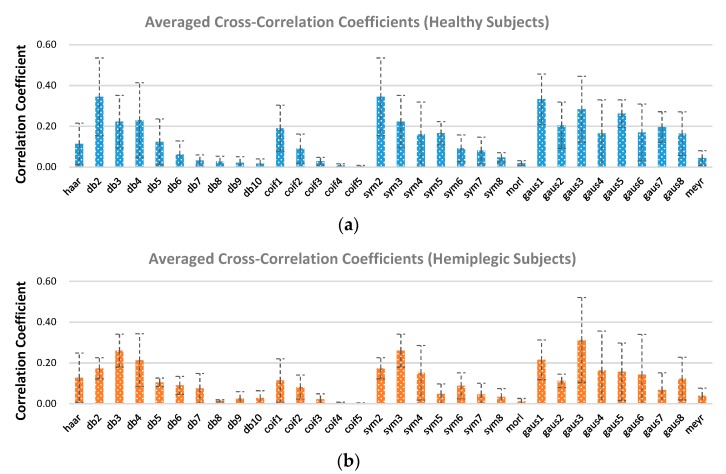
Averaged cross-correlation coefficients between the anterior–posterior knee acceleration signal and specific mother wavelet function for healthy subjects (**a**) and hemiplegic subjects (**b**). Vertical dotted line indicates the standard deviation.

Additionally, the detection of gait events was also performed using two quantitative criteria, the cross-correlation coefficient and the energy-to-Shannon entropy ratio measures, to obtain an appropriate mother wavelet. The analysis results of the mother wavelets based on the cross-correlation coefficient criterion for gait event detection are presented in Figure 8. It can be seen from Figure 8a that amongst the Daubechies family, “db2” and “sym2” achieved the highest average correlation coefficient of 0.34 between the acceleration and the underlying mother wavelet followed by “gaus1” in the Gaussian family with an average value of 0.33. In other words, the “db2”, “sym2”, and “gaus1” mother wavelets have high similarity with the anterior-posterior acceleration signals from which the participants’ gaits were detected. Meanwhile, we found that “db3”, “sym3”, and “gaus3” achieved the best correlation coefficients between the acceleration and the underlying mother wavelet function in their respective categories for the hemiplegic patients (Figure 8b).

**Figure 9:**
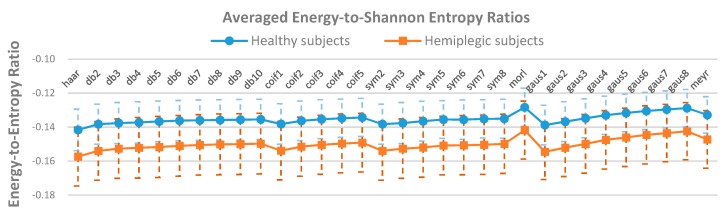
Averaged energy-to-Shannon entropy ratios of CWT coefficients for healthy subjects (blue line) and hemiplegic subjects (red line) across different mother wavelets (*p* > 0.05). Vertical dotted line indicates the standard deviation.

The analysis results of mother wavelet characteristics based on the energy-to-Shannon entropy ratio criterion for gait event detection are presented in Figure 9. It can be observed that the average energy-to-Shannon entropy ratios for most of the investigated mother wavelets lies in the range of −0.15 to −0.13 with the “morl” mother wavelet achieving the highest ratio for healthy subjects (blue line). On the other hand, except for the “morl” mother wavelet that also provided the highest average energy-to-Shannon entropy ratio across the hemiplegic subjects (red line), the other mother wavelets had the average energy-to-Shannon entropy ratios that were in the range of −0.16 to −0.14. Although the “morl” mother wavelet function achieved a relatively higher mean energy-to-Shannon entropy ratio, however there was no significant difference between the “morl” and the other investigated mother wavelet functions based on ANOVA statistical analysis (*p* > 0.05).

## 4. Discussion

Although several CWT-based algorithms have been proposed for gait event detection, the validation of such algorithms especially on hemiplegic patients and the investigation into an appropriate mother wavelet selection has rarely been conducted to date. Thus, there is a need to embark on the current study. This study systematically investigated different wavelet selection criteria across the 32 commonly applied mother wavelets towards obtaining the appropriate mother wavelet required for consistently accurate gait event detection especially for hemiplegic patients. The mother wavelets were individually incorporated into a proposed general CWT algorithm for the recognition of HS and TO gait events, and the algorithm was validated with datasets from hemiplegic patients as well as healthy subjects.

It should be noted that the proposed general CWT algorithm showed a good performance for HS and TO gait event detection in terms of consistently achieving high accuracy and low time-error particularly when the appropriate mother wavelet was applied to both healthy and hemiplegic subjects. The gait event detection results for the healthy subjects were observed to be comparatively stable and consistent with that reported in previous studies which utilized only a specific single mother wavelet [13,22,26]. Interestingly, we also found that the validation results of the estimated gait events for the hemiplegic subjects were observed to be consistent with the results obtained for healthy subjects. One possible reason is due to the use of time-frequency analysis and the domain knowledge about gait events and gait cycles. Since functional gait after stroke is usually affected by spasticity, muscle weakness, and balance disorder [40], hemiplegic gait is often characterized by reduced speed, cadence, stride length and joint angular excursions, and asymmetry in temporal and spatial domains [41]. In addition, limb circumduction is clearly observed on the hemiplegic subjects during walking due to the increased lateral displacement of the foot during the swing in the paretic limb [41], and these factors may increase the complexity of recognizing the gait patterns of hemiplegic patients. However, with the proposed CWT algorithm that incorporates time-frequency information, the gait events of both hemiplegic and healthy subjects can be adequately identified as shown in Figure 3. Therefore, the CWT based algorithm that integrates the optimal wavelet function shows its robustness in HS and TO event detection, which indicates its superiority with no need to readjust the thresholds in comparison with some purely data-driven approaches that are often dependent on threshold and parameter tuning. Hence, this again proves the potential of applying an appropriate mother wavelet to the proposed general CWT algorithm for gait event detection in real-life applications.

When considering the selection of the appropriate mother wavelet, the criteria based on some accuracy and quantitative measures were investigated. For the accuracy-based criteria, time-error and F1-score measures were applied to evaluate the performance of a range of mother wavelets employed by the proposed CWT algorithm to detect the HS and TO gait events. From the analysis results, we found that the “db6” mother wavelet achieved relatively higher F1-scores and yielded the lowest average time-error values for both healthy and hemiplegic subjects. The possible reason is that the “db6” mother wavelet provide the most precise scale for CWT based gait event detection in comparison to the other 31 analyzed mother wavelets. With respect to the quantitative-based criteria, the cross-correlation coefficient (*Xcorr*) and energy-to-Shannon entropy ratio (*ESER*) were investigated to check whether the above two metrics could be considered as effective wavelet selection criteria for the appropriate mother wavelet in detecting gait events. Since the CWT are essentially based on finding the correlation between the analyzed signal (the acceleration signal) and the shifted/scaled mother wavelet, it is obvious that an initial criterion for appropriate wavelet selection could be the cross-correlation (*Xcorr*) that reflects the similarity. However, we found that the results of cross-correlation (*Xcorr*) analysis were not consistent with the actual performances of HS and TO gait event detection in both healthy subjects and hemiplegic patients. This may be due to the fact that cross-correlation (*Xcorr*) is only appropriate for those wavelet-based processing methods based on the resemblance between signals and mother functions [31], but not for our models which extract and distinguish the feature points based on the frequency relationship of the signal itself. After investigating the correlation between the wavelet and the analyzed signal (*Xcorr*), we also wanted to check if there were significant differences that exist in the wavelet coefficients themselves by using different mother wavelets for CWT. Which is to say, the energy concentration and entropy that reflects information lost were integrated to check whether the energy-to-Shannon entropy ratio (*ESER*) could be considered as one of the effective wavelet selection criteria. As pointed out in the previous study [42], maximization of time/frequency energy concentration, minimization of the bias, and the unique relationship that exists between scale and frequency contribute to desirable continuous analytic wavelets. However, we also found that the results of the ESER analysis across different mother wavelets had no significant difference, which might not be appropriate for the wavelet selection in our study. In other words, the results of the *ESER* analysis revealed that the energy concentration and entropy distribution were almost the same across different mother wavelets in the aspect of gait event detection. Therefore, the accuracy-based criteria including time-error and F1-score is suggested as effective wavelet selection criteria in the context of gait event analysis.

Despite the interesting findings observed in this study, there are still some limitations that should be addressed in the future work. For instance, the proposed general CWT algorithm was validated using the dataset from post-stroke hemiplegic patients who were said to be in stages V and IV according to the Brunnstrom assessment scale, excluding patients with severe stroke (perhaps in stages II and III on the Brunnstrom assessment scale). Therefore, there is need to conduct further studies with this population since they have the most drop foot issues, and their gait patterns maybe somewhat different from those in stages V and IV considered in the current study. Besides that, different sensor placement configuration as well as different walking terrains would normally influence gait patterns in real-life applications. In the future, we would further investigate the performances of different mother wavelets in the presence of both factors, and perhaps make adjustment, if necessary, to the mother wavelet selection criteria examined in this study.

## 5. Conclusions

While CWT based gait analysis methods have been widely adopted in previous studies, their performances in gait event detection when using different mother wavelets have rarely been studied, especially in hemiplegic patients. In this study, different mother wavelets and wavelet selection criteria were systematically investigated by using acceleration signals recorded from both hemiplegic and healthy subjects. The experimental results demonstrated that an overall significant difference in performance of the CWT algorithm was observed when using different mother wavelet functions for detecting HS and TO gait events, which suggested the need for this study. Additionally, we found that the accuracy criteria based on time-error minimization and F1-score maximization led to the realization of an appropriate mother wavelet named as “db6” which achieved the highest detection accuracy with lowest detection time-error for both hemiplegic and healthy subjects. The outcomes of this study may provide an insight on mother wavelet selection criteria for gait event analysis especially in hemiplegic patients, and may ultimately facilitate the practical development of rehabilitation devices or strategies for them.

## Figures and Tables

**Table 1:** A brief summary of some previous studies for gait event detection based on wavelet transforms.

Ref.	Subject	Sensor Position	Sensor Type	Wavelet	Detected Gait Parameters
[13]	Healthy subjects	Left and right ankles	Tri-axial accelerometer	Morlet (morl)	HS and TO events
[20]	Healthy subjects	Waist	Tri-axial accelerometer	Gaussian (gaus1)	HS and TO events
[21]	Healthy subjects	Ankle, thigh, waist, chest, upper arm and wrist	Tri-axial accelerometer	Morlet (morl)	HS and TO events
[22]	Parkinson’s disease (PD) patients	Lower back	Tri-axial accelerometer	Gaussian (gaus1)	HS and TO events
[24]	Healthy subjects	Foot, ankle, shank and waist	Tri-axial accelerometer	Daubechies (db2)	HS and TO events
[26]	Healthy subjects	Tibialis anterior muscle of the lower leg	Tri-axial accelerometer	Morlet (morl)	HS and TO events
[27]	Parkinson’s disease (PD) patients	Shank, thigh and lower back	Tri-axial accelerometer	Daubechies (db4)	Freezing of gait

**Table 2:** Clinical and demographic details of the hemiplegic subjects.

No.	Age	Height (cm)	Weight (kg)	State of Illness	Brunnstrom Stage (Lower Limb)	Diagnosis	Symptom
1	59	163	78	12 months	V	Cerebral Ischemic Stroke	Left limb hemiplegia
2	44	171	54	6 months	IV	Cerebral Ischemic Stroke	Right limb hemiplegia
3	53	167	61	7 months	IV	Cerebral Ischemic Stroke	Right limb hemiplegia

**Table 3:** Brief summary of the definitions and main properties of the studied wavelet families.

Wavelet Family	Order N	Orthogonality	Symmetry	Explicit Expression
**Haar**	db 1	Orthogonal	Symmetric	$\psi_{Haar}\left(t\right)=\{\begin{array}{ll} 1 & 0\leq t<1/2\\ -1 & 1/2\leq t<1\\ 0 & otherwise \end{array}$
**Daubechies**	db 2–10	Orthogonal	Asymmetric	No
**Coiflets**	coif 1–5	Orthogonal	Near symmetric	No
**Symlets**	sym 2–8	Orthogonal	Near symmetric	No
**Gaussian**	gaus 1–8	No	Symmetric	$\psi_{Gaussian}\left(t\right)=-\frac{1}{\sqrt{2\pi }}te^{-\frac{t^{2}}{2}}$
**Morlet**	morl	No	Symmetric	$\psi_{Morlet}\left(t\right)=\frac{1}{\sqrt[{4}]{\pi }}e^{i2\pi f_{c}t}e^{-\frac{t^{2}}{2}}$
**Meyer**	meyr	No	Symmetric	$\psi_{Meyer}\left(f\right)=\{\begin{array}{ll} \sqrt{2\pi }e^{i\pi f}\sin\left[\frac{\pi }{2}v\left(3\left|f\right|-1\right)\right] & \frac{1}{3}\leq \left|f\right|\leq \frac{2}{3}\\ \sqrt{2\pi }e^{i\pi f}\cos\left[\frac{\pi }{2}v\left(\frac{3}{2}\left|f\right|-1\right)\right] & \frac{2}{3}\leq \left|f\right|\leq \frac{4}{3}\\ 0 & \left|f\right|\notin \langle \frac{1}{3},\frac{4}{3}\rangle \end{array}$

N is the order of the mother wavelet. ψ is the mother wavelet function if the wavelet has the explicit expression. $f_{c}$ is the wavelet central frequency, t denotes the time, and f denotes the frequency.
